# Supplementary material for: The Extraordinary Case of a Woman with a 30-Year-Long Diffuse Leishmaniasis Cured with One Single Ampoule of Intranasal Pentavalent Antimoniate
Source: Pathogens. 2023 Jun 29;12(7):890. doi: 10.3390/pathogens12070890 (PMC10385054; doi:10.3390/pathogens12070890)
Supplement: Supplementary file 1 [file pathogens-12-00890-s001.zip › pathogens-2448279-supplementary.pdf]

Supplementary Table. Drug susceptibility of parasites isolated from the patient in two occasions. Half-maximal concentration (EC_{50}) and 95% confidence interval.

	**M2269**[a]	**M2506** [b]	**M2874** [c]
	EC_{50} (95% CI) [d]	EC_{50} (95% CI)	EC_{50} (95% CI)
Meglumine antimoniate (g/ml)[e]	391.2 (264.5 – 578.7)	ND[g]	401.4 (339.0 – 475.4)
Amphotericin B (M)[f]	0.034 (0.031 – 0.038)	0.071 (0.062 – 0.081)	0.068 (0.059 – 0.079)
Miltefosine (M)[f]	19.55 (17.61 – 21.70)	47.05 (31.08 – 54.53)	50.54 (46.38 – 55.07)
Pentamidine (M)[f]	5.68 (5.11 – 6.32)	3.86 (3.32 – 4.48)	3.90 (2.67 – 5.69)

[a] Type strain *L. amazonensis* MHOM/BR/1973/M2269. [b] *L. amazonensis* isolate obtained from the patient in June 2008 (MHOM/BR/2008/2506). [c] *L. amazonensis* isolate obtained from the patient in December 2008 (MHOM/BR/2008/2506). [d] 95% confidence interval. [e] Assays performed against intracellular amastigotes. [f] Assays performed against promastigotes.

[g] Not determined.
